# Advances in arthropod-inspired bionic materials for wound healing

**DOI:** 10.1016/j.mtbio.2024.101307

**Published:** 2024-10-22

**Authors:** Yuchen Li, Jiaming Cui, Di Xiao, Bixuan Cao, Jing Wei, Qian Wang, Junwei Zong, Jinwu Wang, Mingzhi Song

**Affiliations:** a School of Pharmacy, Jiangsu Ocean University, Lianyungang, Jiangsu, China; b Department of Orthopedics, Nantong City No. 1 People's Hospital and Second Affiliated Hospital of Nantong University, Nantong, Jiangsu, China; c Liuzhou Traditional Chinese Medical Hospital, Guangxi University of Chinese Medicine, Liuzhou, Guangxi, China; d Department of Orthopedics, the Third Affiliated Hospital of Anhui Medical University, the First People's Hospital of Hefei, Hefei, Anhui, China; e Department of Orthopaedics, the First Affiliated Hospital of Dalian Medical University, Dalian, Liaoning, China; f Department of Orthopaedic Surgery, Shanghai Ninth People's Hospital, Shanghai Jiao Tong University School of Medicine, Shanghai, China

**Keywords:** Wound healing, Biomimetic materials, Arthropod

## Abstract

Arthropods contain lots of valuable bionic information from the composition to the special structure of the body. In particular, the rapid self-healing ability and antibacterial properties are amazing. Biomimetic materials for arthropods have been helpful methods for wound management. Here, we have identified four major dimensions needed to create biomimetic materials for arthropods, including ingredient, behavior, structure and internal reaction. According to different dimensions, we classify and introduce the reported arthropod biomimetic materials. Antibacterial, hemostatic and healing promotion are the main functions of the active compositions of arthropods developed by humans, and most of them play a drug effect. We believe that an ideal biomimetic material of arthropod should have the effect on promoting wound healing through the advantages of structure and composition. The special macroscopic and microscopic structure of the epidermis may provide good mechanical support for biomimetic materials. The drug release regularity in the bionic materials can be referred to the aggressive and secretory behavior of arthropods. The synthesis of substances in arthropods is also noteworthy, and we can learn these special reactions to complete the fast preparation of materials. Arthropod-inspired bionic materials have broad innovation and application prospects in the field of wound repair.

## Graphical abstract

**Figure:**
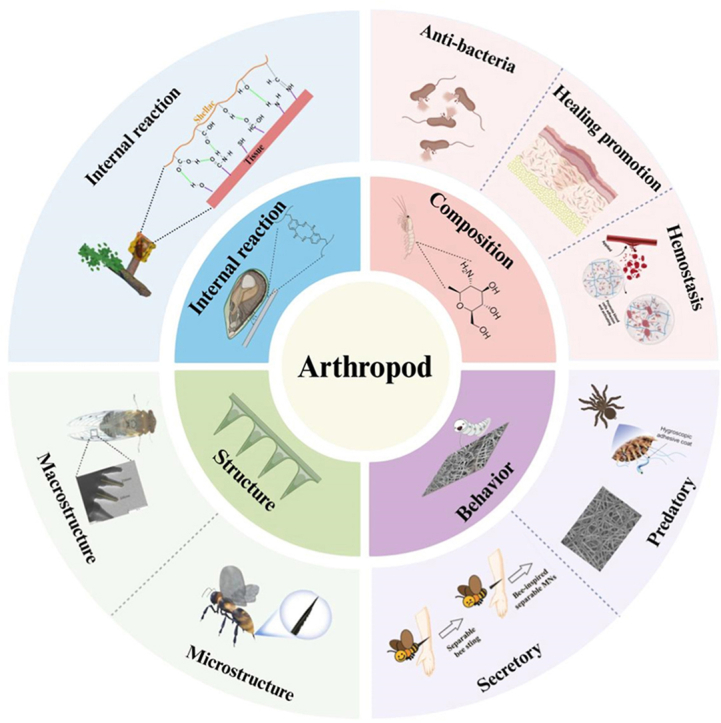

## 1 Introduction

Wounds resulting from trauma, burns, surgical procedures, and chronic diseases exert a profound impact on millions of patients worldwide, with severe cases culminating in disability or even mortality [1]. Additionally, the increasing number of diabetic patients leads to the high incidence of chronic wounds. According to statistics, the annual medical cost of wound treatment in the United States has reached $50 billion, and wound patients spend about $7.5 billion on healthcare [2,3]. This has caused a huge social and economic burden. Wound tissue repair is a complex multi-stage biological process [4]. For acute wounds, bleeding and tissue contamination are the main features. For chronic wounds, the lengthy healing process often goes through three phases including inflammation, proliferation and maturation [5]. In the hemostasis stage, platelets accumulate around the damaged blood vessel wall to form a platelet embolism, meanwhile fibrin forms a blood clot at the wound site. The proliferation stage involves extracellular matrix (ECM) deposition, neovascularization, granulation tissue formation and epithelial formation. The wound would be fully closed at this stage. In matrix remodeling, granulation tissue gradually transforms into scar tissue with few blood vessels at the last stage of wound healing [6]. Therefore, efficacious hemostasis, bacterial inhibition, and tissue regeneration emerge as pivotal functional requirement in biomaterial. Unfortunately, the commonly used wound dressings still have some problems such as poor efficacy and low safety. For instance, collagen and microfibrillar α-cyanoacrylate are conventional medical biomaterials. However, these materials only exhibit general biocompatibility and easily cause infection [7].

In recent years, researchers inspired by the animals to design lots of biomimetic materials with special structures and chemical compositions [8,9]. Most of them have excellent physical properties and medical functions. They have great application potential in the fields of bone repair, wound healing and medical beauty. It is worth noting that arthropod biomimetic materials seem to have developed into a distinct academic field, especially in promoting wound healing. Arthropoda is the largest phylum in the animal kingdom and mainly includes Chelicerata, Crustacea, Hexapoda and Polypoda. The common characteristic of arthropods is segmented body, left and right symmetry, with segmented appendages. They have exoskeleton and open circulatory system. Most arthropods undergo molting and metamorphosis. The familiar flies, silkworms, bees, barnacles, spiders, crabs, barnacles and other organisms belong to Arthropoda. As an ancient type of animals, arthropods are more adaptable to changes in the natural environment than other animals. Their bodies contain enormous amounts of bionic learning information. Flies that live in harsh environments are rarely infested with bacteria. Baby silkworms can spin themselves a tough sleeping bag. A crab that has lost a limb will regrow the same chelicera as the original. The above phenomena illustrate that they have robust defense mechanisms and intrinsic self-repair capabilities over their evolutionary history [10]. This ability to repair may be related to the strong antibacterial, regenerative and adhesion properties of substances in the body fluid or secretions of arthropods, such as the insect antimicrobial peptides [11], the silk protein of silkworm [12], the silk glue [13], the barnacle gum [14], and the purple gel of purple gelatine worm [15], etc. The drug release characteristic is an important part of biomimetic manufacturing. Moreover, the release of venom through biting is a special aggressive behavior of arthropods [16]. This could be used to teach us about bionic design for drug release. The structures of arthropods are intricate and functional. The barbs on praying mantis forelimbs can be used to anchor the prey firmly [17]. This provides important references for bionic learning in material adhesion. The synthesis process of the arthropods’ epidermis is also noteworthy. This unique and rapid curing reaction has been mimicked and used in the preparation of bioadhesives [18]. According to the above, the characteristics of arthropods are very suitable for the design and preparation of wound repair materials.

**Fig. 1:**
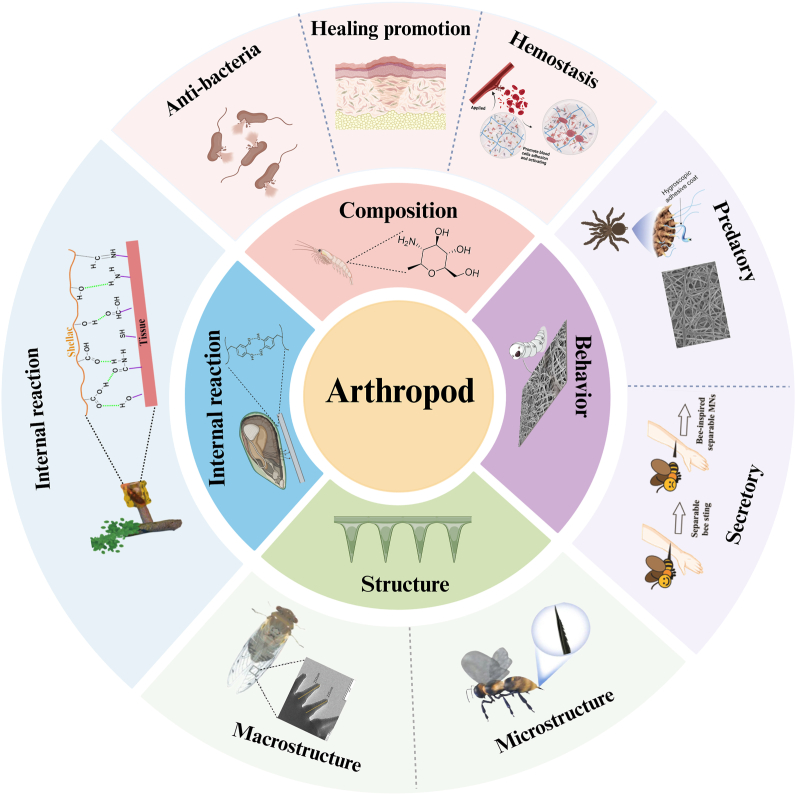
Schematic design scheme of arthropod-based biomimetic materials for wound healing. Created with BioRender.com.

There has been considerable research on biomimetic materials inspired by arthropods, particularly in the context of wound healing. Materials inspired by the natural compositions or synthetic analogs of arthropods, which mimic their structural and behavioral characteristics, are known as arthropod-biomimetic materials. These materials are primarily applied to promote human tissue regeneration. However, despite these advancements, there is a lack of classification based on species-specific characteristics, which has significantly hindered the systematic development of such biomimetic materials. Here, we will review the published biomimetic studies and summarize the different dimensions of research and development work for arthropod biomimetic materials. The following content will be divided into four parts: composition, structure, behaviour, and internal reaction (Fig. 1).

## 2 Arthropod active composition bionics

**Fig. 2:**
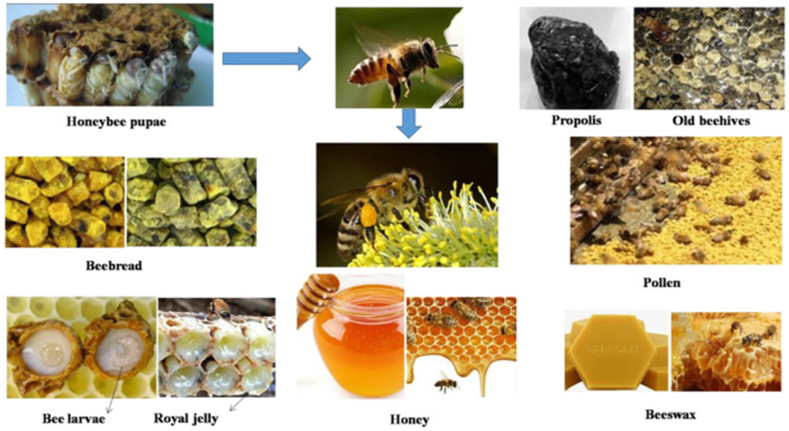
Natural ingredients from honey [19]. Copyright @ 2021 Luo, Dong, Gu, Zhang and Ma.

The secretions or extracts of arthropods play different roles, and the application of active substances into bionic materials has become one of the simplest and most effective bionic ideas (Fig. 2). Arthropods and their secretions have great potential to promote the future development of natural product medicines (Table 1). In this subsection, it will be elaborated according to the different functions played by the active ingredients in bionic materials.

**Table 1:** Arthropodan secretions and compositions.

Product	Components	Effect	Applications	References
Honey	Methylglyoxal, defensin-1, peptide lysozyme, glucose oxidase, phenolic acids	Antibacterial effect	Wounds, burns, ulcers	[[20], [21], [22]]
	Quercetin, naryngenin, kaempferol, chrysin	Anti-inflammatory effect	Wounds, gum inflammation	[20,23,24]
	Carbohydrates, fruit acids, trace elements	Regenerative effect	Wounds	[25,26]
Propolis	Caffeic acid, quercetin	Anti-inflammatory effect	Wounds	[27,28]
	Pinocembrin, galangin, caffeic acid	Antibacterial effect	Wounds	[27,29,30]
	Chrysin	Analgesic effect	Wounds	[31]
	Genistein	Angiogenic effect	Diabetic wounds	[32]
Royal jelly	3-10-dihydroxydecanoic acid, amino gamma globulin	Anti-inflammatory effect	Atopic dermatitis, hyperkeratosis	[33]
	10-hydroxydecanoic acid, 3-10-dihydroxydecanoic acid	Immunomodulatory effect	Autoimmune inflammatory diseases	[33]
Bee wax	Squalene, 10-hydroxy-trans-2-decenoic acid, chrysin	Antibacterial effect	Wounds, atopic dermatitis, psoriasis	[34]
Bee venom	Melittin, adolapin melittin	Anti-inflammatory effect	Plaque psoriasis, wounds, atopic dermatitis	[34]
	Melittin, apamin	Antimicrobial effect	Wounds, acne	[35,36]
Maggot secretions	Maggot proteases, maggot glycosidases	Debris digestion effect	Wounds, acne	[37]
	Maggot lucifensin, maggot seraticin	Antibacterial effect	Wounds	[37]
Cantharidin	Monoterpene	Anticancer effect	Liver cancer, colorectal cancer, bladder cancer	[38]
Silk proteins	Alloferon	Antiviral efffect	Tissue engineering and drug delivery	[38]
*Bombyx mori* hemocyte extract	Not reported	Anti-inflammatory effect	Wounds, periodontitis	[39]
Silk cecropins	Cecropin A	Antiviral effect	Wounds	[40]
Spider silk	Glycine, alanine, serine	Antibacterial effect	Wounds	[41]
Spider venom	Lycotoxins I, lycotoxins II	Antibacterial effect	Bacterial infection	[42]

### 2.1 Active composition for anti-bacteria

**Fig. 3:**
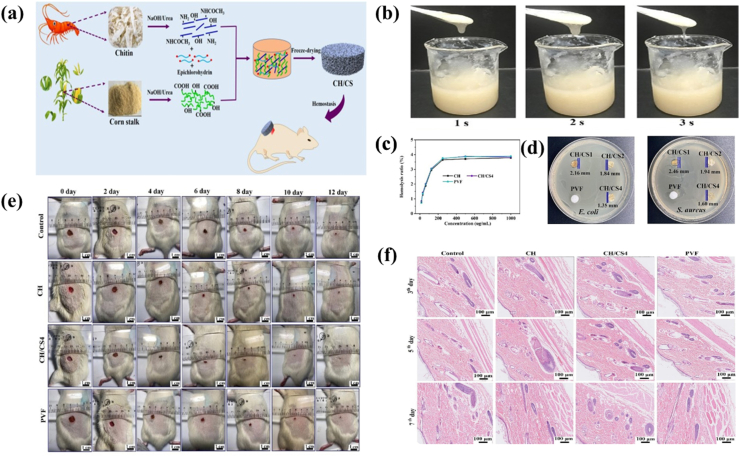
Schematic diagram of CH bionic hemostatic sponge. a) The preparing schematic illustration of corn stalk modified CH composite hemostatic sponge. b) Viscosity changes with time of CH/corn stalk (CS4). c) The hemolysis ratio of CH, CH/CS4 and commercial polyvinyl fluoride sponge (PVF). d) The antibacterial activity of CH, CH/CS1, CH/CS2 and CH/CS4 against *E. coli* and *S. aureus*. e) Photographs of wounds changes with different day for control group, CH, CH/CS4 and PVF. f) The H&E staining of skin tissue treated by control group, CH, CH/CS4 and PVF on the 3rd, 5th, 7th day [48]. Copyright 2023, Elsevier.

Some natural secretions and extracts obtained from arthropods, such as honey [43], chitin (CH)[44], spider proteins [45]and silk proteins [46], have shown good antimicrobial properties. CH present in the epidermis has a strong inhibitory effect on *Escherichia coli* as well as *Staphylococcus aureus*. Disrupting bacterial cell membranes, interfering with metabolism, inhibiting biofilm formation, and activating the immune system in various ways are the main mechanisms [47]. As the result of one new discovery, CH nanosubstances and chitosan (CS) isolated from lobster were also good biological materials (Fig. 3).

**Fig. 4:**
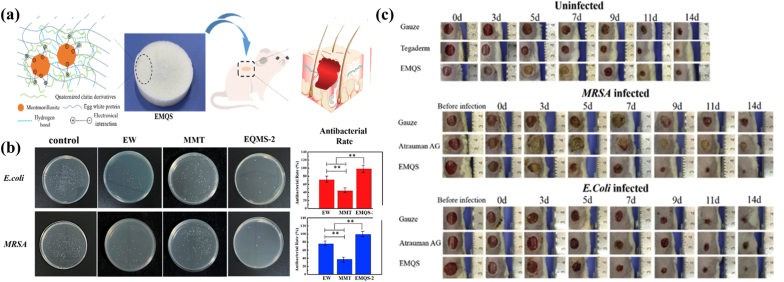
Schematic diagram of composite bionic foam. a) Schematic illustration of the preparation and in-vivo application of an all-natural substances based sponge (EMQS). b) The antibacterial activity evaluation including white protein (EW), montmorillonite (MMT) and EMQS-2. c) Macroscopic changes of the wounds covered with gauze, commercial dressings and EMQS (∗p < 0.05, ∗∗p < 0.01, ∗∗∗p < 0.001) [50]. Copyright 2022, Elsevier.

CH was used in the preparation process of bio-nanocomposites thin films with strong antimicrobial and pro-healing effects. Nanofibrous films prepared based on this material dimension showed excellent resistance to *Aspergillus niger* [49]. In addition, a new CS sponge has also entered the view (Fig. 4). It composed of ovalbumin, quaternised CH derivatives and montmorillonite was designed to shorten the healing time of wounds infected with *Escherichia coli* and methicillin-resistant *Staphylococcus aureus* [50]. Arthropods’ proteins and polysaccharides have already been studied for their antimicrobial activity. For example, *Periplaneta americana* (*P. americana*) has been used in traditional Chinese medicine for a long time [[51], [52], [53]]. *P. americana*–derived drugs, such as *P. americana* polysaccharide (PAP) exhibits strong antibacterial and antioxidant activities [54]. A composite membrane can be prepared by mixing American cockroach CS with this material. The results demonstrated that the strong inhibitory effect of PAP-containing composite membranes on bacteria may be due to the fact that PAP contained many positively charged amino sugars that could bind to cell membranes and cause microbial death [49]. Honey is rich in fructose, flavonoids, polyphenols, and methylglyoxal, and exerts strong antibacterial effects due to pH-altering and high osmotic pressure properties [55]. Researchers designed an alginate hydrogel encapsulating honey to carry out tests. They found that a hydrogel with 4 % honey concentration had the greatest antibacterial activity and the fastest wound healing rate [56]. Silk secreted by the domestic silkworm has a high purity of sericin protein, which has a high content of two protease inhibitors. They inhibit the growth of fungi such as *Ca22q11ndida albicans* and *Coccidioides albicans* by binding to the polysaccharide fractions of the fungal cell wall or by inhibiting the proteases secreted by the fungus [57]. The efficient antibacterial dressings for wound repair can be designed and produced by using green and natural silk as the base material [58]. Their antibacterial mechanism is to kill *Escherichia coli*, *Staphylococcus aureus* and methicillin-resistant *Staphylococcus aureu*s by generating large amounts of reactive oxygen species under light. Meanwhile, the clinical study have shown that the film played an effect on removing bacteria and controlling infections for burn wounds [59]. And the three-dimensional scaffolds prepared by combining natural silk fibroin (SF) with polyurethane showed a strong inhibitory effect on Gram-negative bacteria with the SF concentration of 8 mg/ml [60]. Additionally, silk gel with CS by cross-linking also demonstrated strong inhibitory effect against Gram-negative bacteria [61]. Spider silk is a naturally occurring protein secreted by spiders via their silk glands. It has antimicrobial activity as well as supports the growth of keratinocytes and fibroblasts [62,63]. A range of spider silk bionic composites have also been designed and showed a broad-spectrum antibacterial effect. The hydrogels mixed with hyaluronic acid and spider silk proteins were made by 1-ethyl-3-(3-dimethylaminopropyl)-carbodiimide/N-Hydroxysuccinimide cross-linking method and played a role of anti-Gram-positive and anti-Gram-negative bacteria [64]. The antibacterial effect of spider silk protein hydrogel can be increased by using transgenic technology. PNSR32 and pNSR16 protein tags have already been found and used as the inserting gene sequences to enhance the expression of original spider silk proteins. Wound dressings containing this ingredient can be used for the treatment of second-degree burns in rats. Their excellent antimicrobial properties are related to inserting antimicrobial gene sequences into the sequence of spider silk [65]. Therefore, on the basis of the original function, the antibacterial effect can be improved and strengthened through genetic engineering technology.

### 2.2 Active composition for hemostasis

**Fig. 5:**
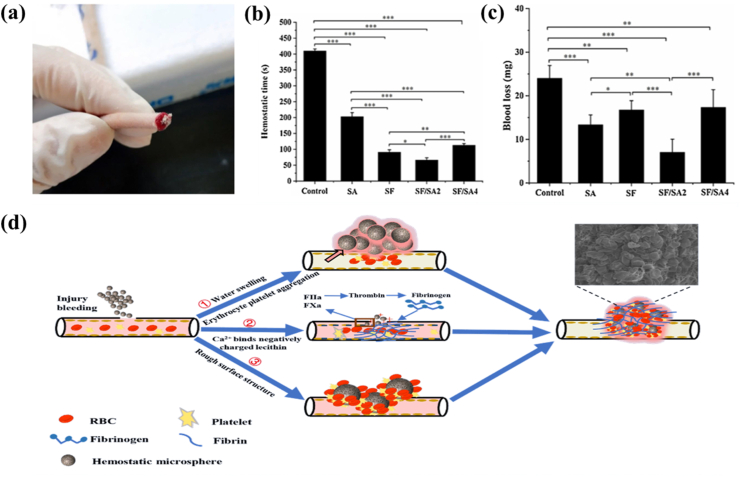
Schematic design of bionic hemostatic microspheres. a) Hemostatic effect of different microspheres on rats. b) Microspheres for hemostasis of tail vein in rats. c) Clotting time of different microspheres in rat tail. d) The coagulation process of the microsphere SF/SA2 includes the physical process of water absorption and swelling of the microsphere, the aggregation of red blood cells and platelets on the surface of the microsphere, and the biological process of Ca^2+^ activating the exogenous coagulation system and activating the fibrinogen into fibrina (∗p < 0.05, ∗∗p < 0.01, ∗∗∗p < 0.001) [73]. Copyright 2021, Elsevier.

Shellac, a natural resin secreted by purple gum insects following sap absorption from host trees, comprises a complex mixture of polyesters and monoesters [66]. Its excellent film-forming and adhesive properties and high biocompatibility compared to other natural biomolecules allow the design of shellac as a hemostatic dressing [67]. Utilizing purple cordyceps as a foundation, a novel wound dressing can be aerosolized to form a mucosal layer within 70 s, achieving rapid haemostasis with notable advantages in ease of application and effective sealing [15]. Shellac can also be added to the preparation of fast-setting liquid bio-bandage, which has broad application prospects such as wound surfaces and limb cross-sections. This bandage can be rapidly set to achieve haemostasis and degrade within a week [68]. Flies, abundant in nature, contain active composition that can promote blood clotting. Some researchers have also prepared a CS sponge derived from fly larva epidermis and applied it to a liver bleeding model in rats. The final result indicated this fly-inspired material could achieve rapid haemostasis by accelerating local cell aggregation, platelet activation, and thrombin generation, compared to the common commercial dressings [69]. Based on emulsion cross-linking method, hemostatic microspheres with different surface roughness would be prepared by using SF and sodium alginate (SA) (Fig. 5). Both in vitro and in vivo coagulation assays showed that the addition of SF to the material increased the coagulation activity and cell adhesion to shorten the bleeding time and reduce the bleeding volume [70]. Nowadays, gel sponges (SP-PEG sponges) prepared from polyethylene glycol (PEG) and SF also demonstrated exemplary hemostasis. Compared to gelatin sponges, SP-PEG sponges were significantly better in reducing haemostasis time and blood loss in a rabbit liver wound model. The mechanism was considered to be that SF significantly activated platelets during blood coagulation, enhanced platelet aggregation and adhesion, and promoted blood coagulation [71]. SF modification has also been tried for the preparation of coagulation materials. Recent experimental results showed that methacrylate-modified SF sealants exhibited superior adhesion, hemostatic, and wound healing properties both in vitro and in vivo, delivering robust and enduring sealing effects [72].

### 2.3 Active composition for healing promotion

**Fig. 6:**
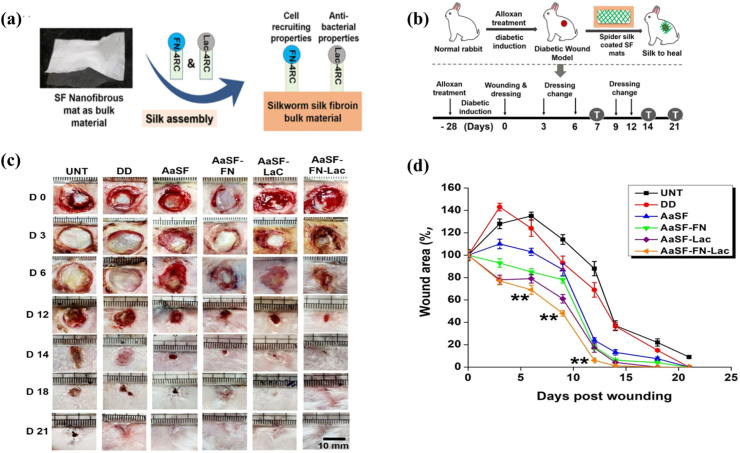
Schematic diagram of silk bionic fiber. a) Schematic representation of the experimental design depicting. b) Methodology to fabricate bioactive silk dressings by coating spider silk fusion proteins on top of SF nanofibrous mats and strategy of treating cutaneous wounds in a diabetic rabbit model using silk dressings. c) Representative gross images of wounds showing wound morphology by different treatments at various time-points during the course of diabetic wound healing. d) Graphical representation of wound area at various time-pointscalculated using ImageJ software demonstrating wound closure rate by different treatments (∗∗ represents p ≤ 0.01) [81]. Copyright 2019 American Chemical Society.

Silk is a long fiber formed by silkworms when their secretions solidify during cocooning [57], consisting of two proteins: silk Fibroin (SF), which makes up about the main structural element of the cocoon, and silk sericin (SS), which binds the sericin filaments to each other, accounting for about 30 % of the cocoon [74]. SF, a widely acknowledged biocompatible tissue protein, has been demonstrated to stimulate collagen activation and production, facilitating wound repair in vivo [75,76]. This satisfactory repair effect is related to the promotion of the attachment and proliferation of human skin fibroblasts and keratinocytes [75]. Compared to conventional gauze, dressings fabricated from SF exhibited superior wound-healing promotion [77]. The SF film prepared by green chemical method could effectively shorten the healing time of total skin defect wounds in rabbits with better skin regeneration compared to commercially available normal dressings. This result was consistent with a randomized, single-blind, parallel-controlled clinical trial on 71 patients [78]. Currently, it is possible to mimic the skin structure of a human using only SF and SA. This bilayer scaffold has been used to help whole-layer wound healing in rabbits [79]. Another SF composite hydrogel was developed for rapid healing of diabetic wounds and demonstrated excellent biocompatibility. It could stimulate fibroblast migration and control oxidative stress in vitro. These effects contributed to tissue re-epithelialization and wound repair in a diabetic type I rat model [80]. Genetic engineering techniques have enabled the presentation of transgenic silkworms expressing human epidermal growth factor (hEGF). Harvesting SF containing hEGF protein for skin dressing preparation has demonstrated enhanced wound healing efficacy [81]. It is well known that arthropods produce two of the most renowned fibrous materials in nature: one is the previously mentioned silk from silkworms, and the other is spider silk. Sakuso et al. used two compositions including antheraea assama silk fibroin (AASF) and functionalized spider silk coating as raw materials to make nanofiber membranes [82] (Fig. 6). AASF provided the basis for cell adhesion and proliferation. For this reason, fibroblasts, keratinocytes and endothelial cells could proliferate and adhere to the fibers rapidly, thus playing the best effect on healing [82]. Additionally, the collagen based dressing film mixed with hydroalcoholic extract of Brazilian propolis promoted differentiation of myofibroblasts as well as greatly increased the epithelialisation rate [83]. The effect of promoting healing could be observed by staining tissue sections from the second week after treatment [83]. The latest study introduced a novel kirigami spider fibroin-based microneedle triboelectric nanogenerator (KSM-TENG) patch [84]. Spider fibroin has been utilized in the fabrication of microneedles due to its sufficient mechanical strength and puncture capability. The KSM-TENG patch demonstrated outstanding performance in reducing the inflammatory response and accelerating wound healing in murine models [84]. Photographic evidence showed that wounds in the microneedles(MNs)-TENG-drug group were nearly healed after 9 days, displaying a significantly higher closure rate compared to both the MNs-drug and control groups [84]. Additionally, spider fibroin exhibited remarkable pro-healing effects and mechanical properties in this study [84].

In this subsection, the different functions played by the active composition in bionic materials was displayed in Table 2.

**Table 2:** The active composition in bionic materials.

ComponentComponents	Inspiration	Material type	Efficacy	References
SF solution (4 wt%)	Silkworm secretions	Films	Keep moisture, inhibit bacteria, accelerate wound healing	[78]
SF solution (10 wt%)	Silkworm secretions	Films	Promote cell proliferation, accelerate wound healing	[85]
SS powder	Silkworm secretions	Films	Promote cell proliferation, increase the production of collagen, scavenge free radical, raise antioxidation ability	[86]
A. assama SF/*B. mori* SF	Silkworm secretions	Hydrogels	Promote cell proliferation, promote angiogenesis, promote epidermal regeneration, accelerate wound healing	[87]
SF solution/Rhein	Silkworm secretions	Hydrogels	Promote cell proliferation, promote angiogenesis, inhibit bacteria, accelerate wound healing	[88]
SF solution/Antioxidant melanin/Therapeutic berberine	Silkworm secretions	Hydrogels	Promote cell proliferation, promote angiogenesis, induce collagen deposition, inhibit bacteria, accelerate wound healing	[80]
SS solution/Carboxymethyl cellulose	Silkworm secretions	Hydrogels	Inhibit oxidative damage, inhibit bacteria, induce collagen deposition, promote angiogenesis, accelerate wound healing	[89]
SF solution/Sodium alginate solution	Silkworm secretions	Scaffolds	Promote cell proliferation, facilitate hemostasis	[79]
SF solution	Silkworm secretions	Scaffolds	Promote cell proliferation, facilitate hemostasis	[90]
SS solution/GelMA	Silkworm secretions	Scaffolds	Promote cell proliferation, keep moisture, accelerate wound healing	[91]
SS solution/Polyvinyl alcohol (PVA)	Silkworm secretions	Scaffolds	Promote cell proliferation, promote collagen synthesis, promote angiogenesis, reduce pain, accelerate wound healing	[92]
SF solution/PEG	Silkworm secretions	Sponges	Promote cell proliferation	[93]
SS solution/CMC solution/Halloysite/Silver nitrate powder	Silkworm secretions	Sponges	Inhibit bacteria, promote angiogenesis, facilitate hemostasis, accelerate wound healing	[94]
SS solution/AgNO_3_/Curcumin/CS	Silkworm secretions	Sponges	Promote cell proliferation, keep moisture, inhibit bacteria, promote angiogenesis, accelerate wound healing	[95]
Collagen/CS	Shrimp and crab shells	Sponges	Induce collagen deposition, accelerate hair follicle repair, form sebaceous gland	[96]
CS/PVA	Shrimp and crab shells	Hydrogels	Facilitate hemostasis, promote cell proliferation, inhibit bacteria	[97]
CS/Gelatin	Shrimp and crab shells	Scaffolds	Keep high porosity, exhibit good tensile strength, maintain biocompatibility	[98]
CS/Poly caprolactone fiber	Shrimp and crab shells	Scaffolds	Accelerate wound healing	[99]
Tualang honey/PVA/CS	Bee secretions	Hydrogels	Inhibit bacteria, keep good swellability, reduce inflammation, accelerate wound healing	[35]
PVA/CS/Honey/Clay	Bee secretions	Hydrogels	Inhibit bacteria, maintain biocompatibility	[100]
Honey/PVA	Bee secretions	Hydrogels	Inhibit bacteria, promote cell proliferation, enhance cell adhesion and migration	[101]
Manuka Honey/Poly (ɛ-caprolactone) (PCL)/Methylcellulose (MC)	Bee secretions	Nanofiber mats	Inhibit bacteria, enhance cell adhesion and migration	[102]
Honey/CS/PVA/Gelatin	Bee secretions	Hydrogels	Exhibit good mechanical properties, inhibit bacteria, maintain biocompatibility	[103]
CS/Gelatin/Honey	Bee secretions	Hydrogels	Inhibit bacteria, accelerate wound healing, accelerate hair follicle repair	[104]
PVA/Sucrose and PVA/Honey	Bee secretions	Hydrogels	Inhibit bacteria, enhance cell adhesion	[105]
Propolis/PEGDA	Bee secretions	Hydrogels	Inhibit bacteria, maintain biocompatibility	[106]
Shellac/Gelatin/Poly (N-isopropylacrylamide)	Purple worm secretions	Nanofiber mats	Accelerate wound healing	[107]
Shellac/PEG/N-octacosanol gallate ester (GA-C28)	Purple worm secretions	Dressinsg	Facilitate hemostasis, inhibit bacteria, accelerate wound healing	[15]
*P. americana* remnant chitosan (PAC)/Polysaccharides (PAP)	American cockroach secretions	Nanofiber mats	Show degradable properties, inhibit oxidative damage	[49]
The recombinant spider proteins pNSR-16 and pNSR-32	Recombinant Spider Silks	Dressings	Reduce inflammation, maintain biocompatibility, accelerate wound healing	[65]
AASF/Recombinant Spider Silk	Recombinant Spider Silks	Nanofibrous mats	Promote granulation tissue development, accelerate wound healing	[81]
AASF/Recombinant Spider Silk	Recombinant Spider Silks	Scaffolds	Promote angiogenesis, promote epidermal regeneration	[82]

## 3 Arthropod behavioral bionics

Understanding the behaviors of arthropods is pivotal for the development of biomimetic materials. These behaviors mainly encompass feeding, secretion, excretion, molting, and eclosion. Inspired by these unique behaviors researchers can design biomimetic materials that promote healing through dynamic 4D effects.

### 3.1 Inspired by aggressive behavior

**Fig. 7:**
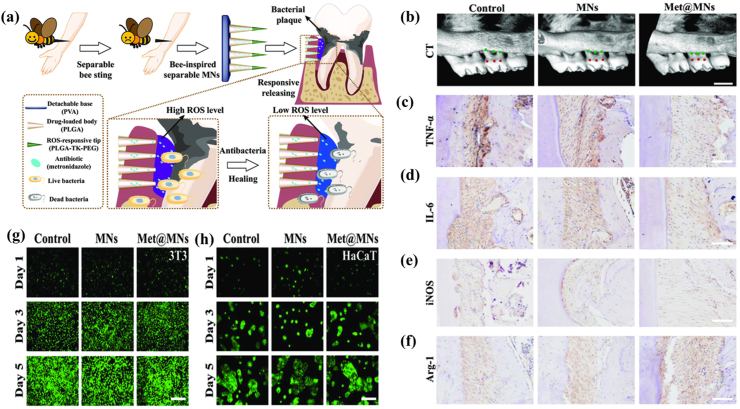
Device and manufacture of the bio-inspired MNs. a) The ROS-responsive MNs tips can be unlocked in the periodontitis status with high ROS levels, leading to increased drug concentration in the gingival sulcus. b) The micro-CT of different treatments. c-f) The representative IHC staining images of periodontal tissue, including TNF-α(c), IL-6(d), iNOS(e), and Arg-1(f). g-h) The fluorescence images of (g) 3T3, (h) HaCaT incubated with MNs for different times [16]. Copyright © 2023 Chuanhui Song.

In addition to collecting honey, honeybees are renowned for releasing venom to deter enemies [108,109]. Inspired by the detachment behavior of honeybee stingers, Song et al. designed detachable microneedles loaded with metronidazole (Fig. 7). Its characteristic was responsive to reactive oxygen species, which could be used to deliver the drug into the gingival sulcus to fight against anaerobic bacteria for further treating periodontitis [16]. The honeybee stinger functions by penetrating its target, utilizing its barbed structure to anchor and pull surrounding tissues [110]. Researchers learnt from this trait and prepared the rigid polymer self-interlocking microneedles patches. The microscopic structure was characterized by unidirectional posterior barbs, which were embedded in various elastomer films. This microneedle patch has shown its great potential for drug delivery and wound tissue repair [111]. The behavior of spiders that capture prey through their webs has also received attention. Silver adenine nanofibers were selected to create the spider web-like nano-agent, which could actively attract and capture bacteria through electrostatic interactions and web morphology features. The new bandage made from this material was more effective in treating bacterial infected wounds in mice than the common commercial bandages [112].

Similarly, spider behavior has also inspired the development of innovative biomimetic materials. Building on the principles of spider prey capture, researchers have developed reduced polydopamine nanoparticles (rPDA) doped with copper-based metal-organic frameworks (Cu-MOF)-hydrogel (GEL-MOF-rPDA). This hydrogel network mimicked the function of a "spider web," capable of anchoring and capturing bacteria. Functional nanoparticles like MOFs, which resembled the spider's fangs and venom, could be encapsulated within the hydrogel to destroy bacterial membranes and deliver an antibacterial effect. This bio-inspired synergistic antibacterial mechanism integrated the processes of “spider web capture”, “spider fangs destroying the outer membrane” and “internal killing by spider venom”. It provided an excellent anti-infective effect [112]. The inspiration for these biomimetic designs comes not only from spider behavior but also from the unique physical and chemical properties of spider silk. Spider silk has been a key material of interest due to its exceptional mechanical strength, temperature adaptability, and unique composition. With its high specific strength, excellent elasticity, and remarkable toughness, spider silk fibers outperform many other natural and synthetic fibers [113] (Table 3). These properties are especially important for designing biomimetic materials that need to replicate the flexibility, toughness, or elasticity of biological tissues [114,115]. By understanding and leveraging these traits, researchers have created bio-inspired materials that mimic the spider's attack mechanisms, such as the ability of puncturing, stretching, and holding, enhancing their application in drug delivery and tissue repair.

**Table 3:** A summary of natural and synthetic silk.

Type	Components	Inspiration for the bionics	Mechanism	Physical properties	References
Recombinant spider silk	Recombinant amino acid sequence [(GGX)nFGAILSS]128	Amino acid sequence of spider silk protein	Motif reorganization and polymerization of different folding structures	Tensile strength 0.98 GPa; Toughness 161 MJ/m^3^	[116]
Recombinant spider silk	Recombinant amino acid sequence [An(GGX)n]192	Amino acid sequence of spider silk protein	Protein covalent assembly	Tensile strength 1.03 GPa; Toughness 114 MJ/m^3^	[117]
Recombinant spider silk	Recombinant amino acid sequence NTD-[An(GGX)n]-CTD	Amino acid sequence of spider silk protein	Protein non-covalent assembly	Tensile strength 834 MPa; Toughness 143 MJ/m^3^	[118]
Recombinant spider silk	Recombinant spider silk protein	Amino acid sequence of spider silk protein	Standardized DNA part assembly; Split intein-mediated ligation	Tensile strength 1.03 ± 0.11 GPa; Modulus 13.7 ± 3.0 GPa; Extensibility 18 ± 6 %; Toughness 114 ± 51 MJ/m^3^	[118]
Recombinant spider silk	Recombinant spider silk protein	Amino acid sequence of spider silk protein	Metal ion incorporation; Chemical covalent cross-linking	Toughness 249 ± 22 MJ/m^3^; Tensile strength 6.3 ± 0.7 GPa	[119]
Recombinant spider dragline silk	Water-soluble recombinant spider dragline silk protein (with a low molecular weight of 47 kDa)	Amino acid sequence of spider silk protein	Microfluidic wet-spinning	Tensile strength 510 MPa; Extensibility 15 %	[120]
Hydrogel fiber	Polyacrylic acid and silica nanoparticles	The inner and outer core architecture of natural spider silk	Self-assembly of hydrogel fibre	Tensile strength 895 MPa; Extensibility 44.3 %	[116]
Recombinant spider silk	Bovine serum albumin recombinant spider silk proteins	Amino acid sequence of spider silk protein	Non-covalently crosslinked	Tensile strength 1180 MPa; Toughness 433 MJ/m^3^	[121]
Hydrogel fiber	Sericin and polyacrylamide	Sericin	Covalent cross-linking	Porosity of the samples ranged from 70 % to 83 %; PH-swelling dependence; Low biological degradation	[122]
Supramolecular elastomer	Poly (urethane-urea) polymers	Architecture of natural spider silk	Hydrogen-bonding segments	Toughness 390.2 MJ/m^3^; Tensile strength 75.6 MPa	[123]
Hybrid macrofiber	Inorganic hydroxyapatite (HAP) and PVA	Rigid crystalline and flexible amorphous protein blocks spider of silk fibers	Biomineralization	Toughness 296 ± 12 MJ/m^3^; Tensile strength 949 ± 38 MPa; Stretch ability 80.6 %	[45]
Recombinant spider silk	Recombinant spider silk protein	Amino acid sequence of spider silk protein	Self-assembly of recombinant spidroins	Tensile strength 370 ± 59 MPa; Extensibility 110 ± 25 %; Toughness 183 ± 33 MJ/m^3^	[124]
Artificial fiber	Recombinant spider silk protein	Natural spinning	Microfluidic spinning	Tensile strength 510 MPa; Extensibility 15 %	[125]
Hydrogel fiber	Vinyl‐functionalized silica nanoparticles	Hierarchical core–shell structure	Self-assembly of the polymer chains	Tensile strength 895 MPa; Toughnes 370 MJ/m^3^; Damping capacity 95 %	[126]
CS filament	KOH/urea solution CS	CS	Wet-spinning strategy	Tensile strength 878 ± 123 MPa; Elastic modulus 44.7 ± 12.3 GPa	[127]
Fiber	Recombinant spider silk protein	Amino acid sequence of spider silk protein	Wet-spinning strategy	Toughness 160.1 ± 21.4 MJ/m^3^	[128]

### 3.2 Inspired by secretion behavior

**Fig. 8:**
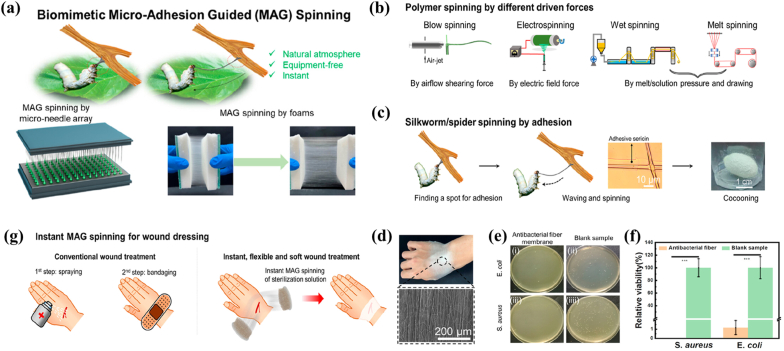
Schematic of the biomimetic microadhesion guided (MAG) spinning. a) General schematic of (MAG) spinning. b) Representative well-developed spinning techniques and their working mechanisms. c) Schematic of silkworm spinning process based on an adhering-pulling-adhering mechanism. d) Digital photo shows the deposition of conformable antibacterial fibers onto the back of the wrist by the MAG spinning and the SEM image of the deposited fibers. e-f) Photographs and statistics of an antibacterial fiber membrane against S.aureus and E.coli. g) Illustration of MAG spinning as an instant, in situ fiber-fabrication technology for wound dressing application (∗p < 0.05, ∗∗p < 0.01, ∗∗∗p < 0.001) [130]. Copyright 2017, Elsevier.

In nature, both spiders and silkworms produce long silk fibers with properties adapted to environmental changes via an efficient and straightforward secretion way [129]. The concept of micro adhesion guided (MAG) spinning arose from authors' observation and mimicry of silkworm behavior (Fig. 8). The head movement of silkworms during the spinning process was learnt to develop three different biomimetic MAG spinning modes including oriented fibers, layered crosslinked fibers, and one-piece fibers. Finally, compliant antimicrobial fabrics would be successfully deposited in situ onto the wound by MAG spinning and rationally designing the fiber composition. This prepared dressing could be used to prevent microbial infections as well as to reduce inflammatory responses [130]. An injectable silk protein bioscaffolds with good porosity and mechanical property were prepared by simulating the processes of natural spider and silkworm silk moulding. This preparation process used the low stress/strain and shear thinning characteristics of the concentrated silk protein solution. The resulting scaffold demonstrated good effects of drug slow release, defect filling and promoting tissue regeneration [131]. Furthermore, microfluidic technology has also been applied to the work of arthropod bionics. Spider's large cystic glands are its silk reeling organ. Large cystic gland microfluidic chip could be mimicked by using aqueous regenerated silk protein and cellulose nanofibers as the basic spinning solution to create novel regenerated silks [132]. These nanofibers hold promise for applications in tissue repair and regeneration, and intelligent electronic components.

## 4 Arthropod structure bionics

Different from other phyla, arthropods exhibit distinctive characteristics in body structure division, appendages, and body walls. These unique features have evolved as adaptive mechanisms to navigate complex environmental challenges and resist external invasions. For imitating arthropods, humans studied these biological structures to construct mechanical devices that mimic either entire organisms or specific parts, achieving analogous functions through structural replication. Currently, significant advancements have been achieved in both macroscopic and microscopic aspects of arthropod structure bionics.

### 4.1 Macro-structural bionics

**Fig. 9:**
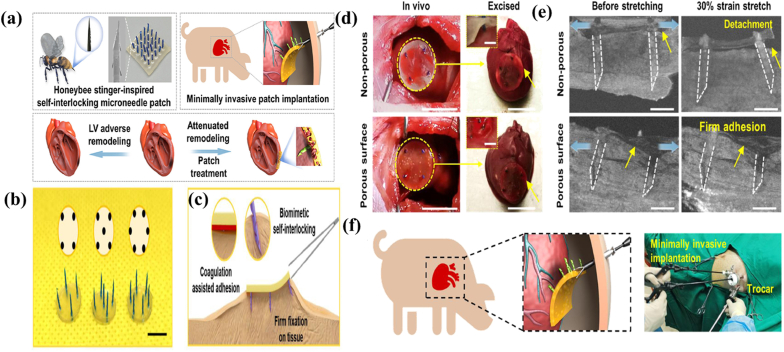
Design of the honeybee stinger-inspired microneedle patch and function. a) Design of the honeybee stinger-inspired of self-interlocking microneedle patch. b) Microneedle patches with different microneedle patterns and numbers. c) Schematic illustration of microneedle patch fixation on soft tissue combining the effect of biomimetic self-interlocking and coagulation assisted adhesion. d) Left: implantation of microneedle patches onto beating rat hearts. Right: status of microneedle patches on excised hearts. e) 3D reconstruction of implanted patches on chicken breast tissue. f) Thoracoscopy-based minimally invasive implantation of microneedle patches onto epicardium in a porcine model [111]. Copyright 2022, Elsevier.

Bee stingers and mosquito mouth needles brought the design inspiration of delivery carriers that minimally invade and sustained-release drug [133,134]. Building upon this, artificial bionic microneedles have been developed [135]. Unsatisfying tissue adhesion and bad transdermal drug delivery are the most obvious drawback of traditional microneedle patches. In response to the above problems, a bee-inspired microneedle has emerged to mimic the barbed structure of the bee stinger (Fig. 9). The comparison results with ordinary microneedles showed it was easier to be inserted into the skin to enhance tissue adhesion and it had better transdermal drug delivery [136]. Mosquito mouthparts comprise a fixed positioning segment and a liquid transfer component. Based on these special structures, the mosquito-inspired microneedle dressing was developed and featured an ultra-fine tip, customizable pattern design, adjustable needle length, and varying mechanical strengths. This design could enable intelligent and painless drug delivery [137]. Mosquito mouthparts, caterpillar spines, Cicadellidae mouthparts, tsetse fly mouthparts, honey bee stingers, and paper wasp stingers are a few examples of insects used in designing MNs' structures [[138], [139], [140]]. With a pagoda-like multilayered structure, the skin lesion healing microneedle patch has also appeared. Its creation was inspired by ladybirds, gadflies and wasps and its high adhesion property was help to facilitate quick haemostasis. Moreover, this microneedle was also able to be loaded with glucocorticoids for treating skin lesion like psoriasis [141]. With the comprehensive observation of natural organisms, researchers have discovered that structure of claws is helpful for the further improvement of both fixation and wound closure in the design of bionic materials. Researchers have designed a microneedle (MN) patch inspired by shark teeth, this MN patch also has a tilted special structure that allows easy penetration of the skin, especially by preserving a stable adhesion during long-term recovery from chronic wounds [142].

### 4.2 Micro-structural bionics

**Fig. 10:**
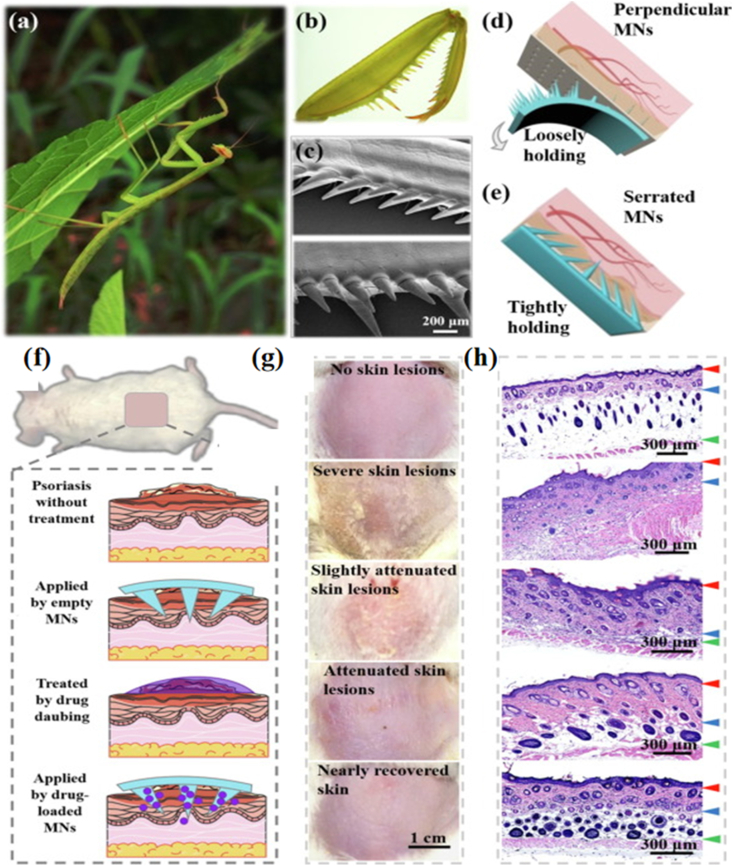
Schematic illustrations of bioinspired serrated clamping MNs. a) Digital image of a mantis climbing on a leaf. b-c) Optical and SEM images of the forelegs of mantises showing slant serrated microstructure. d) Perpendicular MN array: the gray arrow shows it is prone to fall off from the skin when placed upside down. e) Bio-inspired serrated clamping MN array has the ability to hold on firmly. f) Scheme of normal mouse dorsal skin and different treatments for the four groups of imiquimod-induced psoriasis mice. g) Digital images of the corresponding dorsal skin after 3 days of therapy. h) Hematoxylin-eosin staining of dorsal skin after 3 days [17]. Copyright 2019, Elsevier.

By observing the praying mantis tail spines, scientists have discovered that each layer of the tail barbs was characterised by different microstructures and chemical compositions [53]. Mimicking the tail barbed structure of praying mantis, they presented a tip tilted from both sides to the middle snap-like hydrogel microneedle patch. The main raw material for preparation was magnetically responsive magnetic fluid (Fig. 10). This microneedle patch exhibited excellent skin adherence, maintaining attachment even during movement. Such robust adhesion ensured stable performance with minimal skin irritation [17].

Australian scientists have discovered that nano-tips attached to the upper surface of the wings of dragonflies and cicadas. The cell membranes of bacteria would be physically disrupt when they attached to the wing surface. Therefore, these nano-tips have been regarded as a sterilising agent [[143], [144], [145]]. A nano-enzyme with self-assembled tertiary layered structure was developed successfully based on the inspiration above. This enzyme used the surface nanocone topology to physically rupture the bacteria and then catalytically bind to peroxidase-like enzymes, which combined to cause bacterial death. It might meet the requirements of tissue engineering, where its unique antibacterial capacity was expected to become a major function of biomedical materials [146].

## 5 Internal reaction bionics

**Fig. 11:**
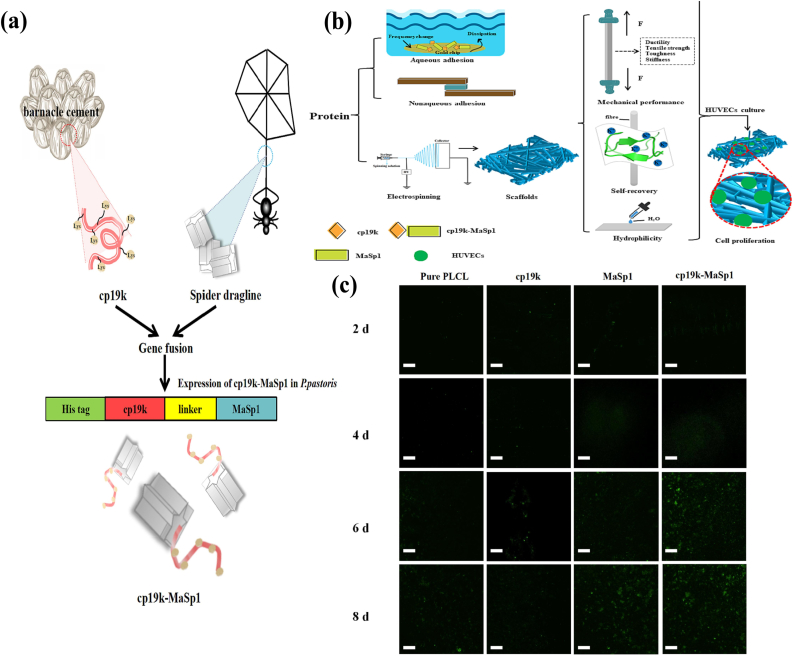
Schematic design of bioinspired synthetic fused protein adhesive. a) A fused protein (cp19k-MaSp1) composed of barnacle cement protein (cp19k) and spider silk protein (MaSp1) was genetically designed. b) Protein scaffolds prepared from protein solutions by electrostatic spinning and biocompatibility, mechanical performance, self-recovery of derivative scaffolds. c) Fluorescence images of the proliferation for HUVECs on different fiber scaffolds [154]. Copyright 2023, Elsevier.

Barnacles are masters of underwater adhesion [147], and by secreting barnacle adhesive proteins, they can firmly adhere to the surfaces of objects [148]. An emerging hydrogel with cationic and hydrophobic amino acid-rich features like those of barnacle mucin could securely adhere to surfaces of various materials (Fig. 11) underwater through interfacial electrostatic and hydrophobic interactions [149,150]. After learning from the experience of barnacle adhesion protein processing in vivo, researchers created an arthropod bionic hydrogel with strong adsorption capacity to wet tissue surfaces, rapid self-healing ability, and excellent antimicrobial properties due to its rich phenolic hydroxyl groups and the dynamic redox balance of phenol quinones. Animal studies have demonstrated its superior wound healing acceleration compared to commercial hemostatic sponges, as well as its efficacy in stemming bleeding from deep wounds [151].

Some researchers have discovered phenol-amine synergy during the formation of the insect exoskeleton. Actually, this rapid and amazing hardening reaction is a special cross-linking between polymers through an oxidation reaction involving phenol-quinone [152,153]. A new type of adhesive was developed. Oxygen in the air and dissolved in water were treated as the initiator and participated in the reaction between phenol and polyamine molecules. The phenolic radicals and quinones generated are involved in the curing process of the strong adhesive. This mimicked the active substances involved in the hardening process of the epidermal shell of insects. Its advantages were eco-friendly and biocompatible strong adhesion. The instantaneous tissue adhesion of this strong adhesive significantly reduced the wound closure time, compared to the closure time of conventional adhesive glues. The other thing the adhesive did was to promote skin regeneration and minimise scar formation [18].

## 6 Discussion

Bionic materials, emulating the sophistication of natural organisms, are increasingly capturing scientific attention. Here, a comprehensive literature review we did revealed that arthropod-derived biomimetic materials were predominantly applicated in wound healing and soft tissue repair. More importantly, these innovations drew inspiration from four primary dimensions: composition, structure, behavior, and internal reactions.

In the theory we introduced, effective composition bionics is usually the easiest and most widely used one-dimensional form of arthropod bionics. By utilizing antimicrobial and pro-healing active substances inherent in arthropods, researchers have solved problems in wound repair such as biocompatibility, wet adhesion and microbial infections [53]. Then, the highly viscous components of arthropods were used to seal wounds and stop bleeding [155]. Due to the overuse of antimicrobial drugs, many bacteria have evolved drug-resistant strains [156]. However, based on bionic manufacturing technology, CH modified by imidazole ring with broad-spectrum antimicrobial activity and high biocompatibility is a good solution to this problem [157]. The liquid biological bandage made of purple worm shellac could quickly stop bleeding and completely degraded within seven days [64]. Glycoprotein extracted from American cockroach accelerated wound healing, collagen synthesis and angiogenesis by promoting the proliferation and migration of keratinocytes/fibroblasts and stimulating the secretion of epidermal cell growth factor and vascular endothelial growth factor [158]. However, single active composition biomimetics can no longer satisfy our daily needs, multi-ingredient biomimetic arthropod materials become a new research and development direction. Researchers have combined two or more new biomimetic proteins or chemically modified components to create dressings and fillers that better facilitate repair [159].

Innovative microneedle designs, inspired by insect mouthparts and barbs, exemplify effective structural bionics [160]. These structurally bionic microneedles, such as snap-gel microneedles mimicking praying mantis caudal stings and pagoda microneedles emulating bee barbs, exhibit superior adherence and enhanced transdermal properties, compared to conventional microneedles. Recent studies demonstrated that these microneedles could overcome tissue barriers and penetrate microbial biofilms, thereby increasing the bioavailability of active substances [161]. Such advancements are pivotal for designing smart sensing microneedles capable of providing real-time feedback on skin inflammation and healing. However, microneedle dressings may not be suitable for extensive burns or chronic wounds, necessitating the development of large-area fiber films utilizing SP for bionic materials. In a few words, structure and composition are two intuitive dimensions of biomimicry. A simple combination of them can be used for the design of two-dimensional arthropod-inspired biomaterials to meet the release of the main composition on demand.

The rich biological characteristics and unique functions of arthropods are also closely related to their own behavior and internal reaction processes [162]. Inspired by nature, researchers have developed microadhesion guided spinning techniques simulating the flexibility and efficiency of silkworm silk production. The design of the latest self-interlocking microneedle patches and potent bactericidal nano-enzymes draws inspiration from the feeding behaviors of bees and spiders. Additionally, the special behaviors of arthropods, such as insect mimetic behavior can be used to design mat responsive multifunctional wound dressings, as well as the courtship behavior of fireflies to design light-responsive materials [163,164]. The arthropods-inspired active substance release of smart wound dressings can be adjusted based on changes in the in vitro environment (UV, NIR, magnetic fields, and ultrasound) or the microenvironment at the wound sites (pH, enzymes, reactive oxygen species, and temperature). These smart wound dressings can improve the healing rate of chronic wounds, effectively reduce the recurrence rate, reduce the pain and economic loss of patients, and have a good prospect in wound dressing. Behavior is the third dimension of bionic design, and its existence makes wound repair materials have responsiveness and intelligence.

Arthropods use special biochemical reactions in the synthesis of secretions and epidermis. For instance, barnacles achieve adhesion to surfaces through the secretion of adhesion proteins rich in phenolic hydroxyl groups and dynamic redox balance of phenol quinones. The unique quinone reaction of the insect epidermis can lead to a fast change in the properties of the epidermis [165,166]. Insect physiological reactions are numerous and have unlimited potential for development. Take the quinone tanning reaction as an example, before and after the quinone tanning reaction, the strength of the insect epidermis is significantly increased [167]. This epidermis is composed of CH and collagen, but current production processes do not allow for increasing mechanical strength of hydrogels prepared using both. This is seriously inconsistent with the exoskeletons that insects have in nature. We can imagine that after successfully imitating the quinone tanning reaction, the biomechanical strength of biomimetic hydrogels will be greatly improved. At the same time, arthropod bionics will also enter a new dimension.

With bionic arthropods, we have remedied the deficiencies in traditional material design and preparation, especially the adhesion properties, antimicrobial effects, hemostatic potency, and biocompatibility. However, these are still unable to match the actual needs of complex wound repair. Through a literature review, we find that most biomimetic materials are still in the stage of single or two dimension biomimetic. This means that the development of wound repair materials is expected to achieve the best functional effect through multi-dimensional combined design. There is no denying that the current design of wound dressings is similar. Many shortcomings in the design of biomimetic materials have presented. The uncertainty of efficacy is caused by the poor active ingredient or the unsatisfactory drug release effect. In addition, a series of side effects and poor adhesion also affected the use of wound treatment materials. Using the four dimensions of biomimetics proposed by us can effectively solve the existing problems. Firstly, most of the arthropod components are safe for humans. Unlike antibacterial compound, biomacromolecules derived from arthropods do not produce organ toxicity such as liver and kidney. At the same time, bionic compositions are not prone to drug resistance. The ability to activate the body's exogenous hemostatic mechanism is a significant advantage of arthropod-derived compositions, enabling them to exert a strong hemostatic effect. Active ingredients that promote tissue regeneration have also been proven and are thought to be useful in designing bionic wound dressings. Another key point is that different species exhibit distinct structural characteristics. Though many arthropods only have flimsy epidermides, but they can withstand impacts far greater than their weight. Reasonable epidermis structure not only makes arthropods become masters of design, but also superior in raw material saving. Ideal mesoporous, handiness, enough elasticity and other parameters are the highlights of arthropod bionic hydrogel. Thirdly, unlike the complex behaviors of other organisms, many behaviors of arthropods can be imitated or even copied. Mimicry of these behaviors could provide valuable ideas for the design of responsive materials. This is critical for the treatment of skin lesions, because the wound repair process often goes through a series of dynamic processes such as hemostasis, antibacterial, anti-inflammatory, angiogenesis, tissue repair, and epithelial coverage. Moreover, changes in the wound microenvironment, such as PH, temperature, and microbial type, are also factors that need to be considered when preparing responsive materials. Therefore, release of different drugs in the same material at different conditions is the base function for an excellent wound material. Last, in materials bionics, some researchers have begun to conduct researches on the synthesis reaction of the internal components of living organisms. This is a positive direction to think in. These special synthetic reactions are not available in mammals, and their advantage is reflected in the low requirements for the type of reaction substrate and the easy availability of catalysts for the reaction process. Quinone tanning reaction is the most convincing representative, because hardness and color changes of composition will be completed in a very short time. We can use these special reaction processes to achieve rapid preparation of wound repair materials. Based on the above description, according to different wound conditions, it is necessary to design biomaterials by fully considering the four dimensions of bionics proposed in this review.

It must be clearly seen that the current research on arthropod bionic materials is still in the early stage. Compositions, structures, behaviors and the substance reaction process in the arthropods' body need to be further explored and discovered.

## 7 Conclusion and perspectives

Biomimetic materials for arthropods have become a new research field. The current application is mainly in skin injury and wound repair. These emerging materials can be used for clotting, anti-bacteria, anti-inflammatory, or pro-tissue repair. The most common bionic species are barnacles, bees, silkworms, spiders, purple worms and cockroaches. The four dimensions of arthropod biomimetic are summarized here, which can facilitate the design and preparation of biomimetic materials. We believe that an ideal biomimetic material of arthropod should have the effect on promoting wound healing through the advantages of structure and composition. The special macroscopic and microscopic structure of the epidermis may provide good mechanical support for biomimetic materials. The drug release regularity in the bionic materials can be referred to the aggressive and secretory behavior of arthropods. The synthesis of substances in arthropods is also noteworthy, and we can learn from these special reactions to complete the fast preparation of materials. Arthropod-inspired bionic materials have broad innovation and application prospects in the field of wound repair.

However, the transition from laboratory findings to clinical applications is fraught with complex challenges, necessitating systematic research and technological advancements. Foremost among these challenges is the assurance of biocompatibility and long-term safety. Rigorous preclinical evaluations and multi-phase clinical trials are essential to rule out potential immunogenic responses and chronic toxicity associated with bioactive compounds and structural elements derived from arthropods. Advanced biomaterial characterization techniques, coupled with comprehensive in vitro and in vivo models, are required to thoroughly understand interactions with host tissues, ensuring clinical efficacy and safety.

Another significant hurdle lies in scalability and production efficiency. The replication of the intricate structures and functions of arthropod-inspired materials often involves sophisticated manufacturing techniques, such as nanotechnology and 3D printing. While these methods have shown promise in small-scale experiments, their application in large-scale production is hindered by high costs and technical challenges. To facilitate commercial and clinical use, it is important to develop scalable and efficient manufacturing processes that optimize material synthesis, reduce costs, and maintain structural integrity and functional performance. Furthermore, the integration of automated and intelligent manufacturing platforms could enhance production efficiency, ensuring consistency and reproducibility of these advanced materials.

Accurately replicating the complex micro- and macro-structures of arthropods is meaningful to achieving the desired mechanical properties and biological functions of biomimetic materials. This requires not only state-of-the-art manufacturing technologies but also interdisciplinary collaboration across material science, biology, and engineering. In the dynamic environment of wound healing, these biomimetic materials must demonstrate sufficient stability and adaptability to cope with varying physiological conditions, such as changes in humidity, pH, and mechanical stress. We need to investigate the performance of these materials under different circumstances to ensure their practical effectiveness.

Looking ahead, as the tendency of wound treatment gradually transitions towards personalized and precision medicine, the development of intelligent therapeutic solutions presents transformative potential to revolutionize wound care strategies. The integration of biomimetic materials inspired by arthropods with stimuli-responsive hydrogels is hope to meet the demands in personalized and precision medicine. These smart hydrogels can respond to various external stimuli, exhibiting functions such as stimulus-induced expansion, contraction, and bending, thereby facilitating extensive applications in areas like smart drug delivery and actuators. Typical stimuli include temperature, pH, and reactive oxygen species (ROS). Currently, maggot therapy is increasingly applied in wound healing [168]. The advantage of this treatment is self-evident, but its disadvantage is that it is easy to cause patients fear. According to the design dimensions of the arthropod bionic material proposed in this paper, many unexpected advanced materials can be designed and prepared. A 4D bioinspired smart hydrogel, mimicking the macro- and microstructures of maggots for enhanced wound surface adhesion is achievable. This hydrogel could autonomously respond to environmental temperature changes and programmed release of the bioactive substances imitating maggots’ secretion. As a more patient-friendly alternative, the hydrogel would accelerate the release of therapeutic agents when the wound surface temperature rises during the early stages of healing, thereby promoting wound repair.

Concurrently, the combination of arthropod-inspired biomimetic materials with smart medical technologies offers bright prospects for wound healing. However, their underlying mechanisms mainly involving hemostasis, antimicrobial activity and tissue regeneration require further exploration. By integrating interdisciplinary insights from molecular biology, materials science, and bionics, researchers are poised to elucidate the critical biological processes, including cell migration, inflammatory responses, and angiogenesis, which are regulated by these materials. If the unique advantages of arthropod-inspired biomimetic materials can be coupled with advanced real-time monitoring and adaptive feedback sensing technologies, they are likely to optimize therapeutic strategies, enhance healing efficiency, and reduce infection risks. These groundbreaking innovations promise not only to improve patients' quality of life but also to propel materials science into a new era, paving the way for broader medical applications and laying the foundation for the next generation of medical technologies.

## Funding

This study was supported by the 10.13039/501100001809National Natural Science Foundation of China (82204822/82104864) and the 10.13039/501100002858China Postdoctoral Science Foundation (2023M732315).

## CRediT authorship contribution statement

**Yuchen Li:** Writing – original draft, Validation, Project administration, Formal analysis, Conceptualization. **Jiaming Cui:** Validation, Resources, Investigation, Formal analysis, Data curation. **Di Xiao:** Writing – original draft, Project administration, Formal analysis, Data curation. **Bixuan Cao:** Resources, Methodology, Investigation, Data curation. **Jing Wei:** Resources, Methodology, Investigation, Formal analysis, Data curation. **Qian Wang:** Data curation. **Junwei Zong:** Funding acquisition, Supervision, Validation, Writing – review & editing. **Jinwu Wang:** Writing – review & editing, Project administration, Investigation, Funding acquisition, Conceptualization. **Mingzhi Song:** Writing – review & editing, Writing – original draft, Validation, Project administration, Funding acquisition, Conceptualization.

## Declaration of competing interest

The authors declare that they have no known competing financial interests or personal relationships that could have appeared to influence the work reported in this paper.

## Data Availability

No data was used for the research described in the article.
